# Involvement of unconventional myosin VI in myoblast function and myotube formation

**DOI:** 10.1007/s00418-015-1322-6

**Published:** 2015-04-21

**Authors:** Justyna Karolczak, Iuliia Pavlyk, Łukasz Majewski, Magdalena Sobczak, Paweł Niewiadomski, Yuriy Rzhepetskyy, Agata Sikorska, Natalia Nowak, Paweł Pomorski, Tomasz Prószyński, Elisabeth Ehler, Maria Jolanta Rędowicz

**Affiliations:** Department of Biochemistry, Nencki Institute of Experimental Biology, 3 Pasteur St., 02-093 Warsaw, Poland; Department of Cell Biology, Nencki Institute of Experimental Biology, 3 Pasteur St., 02-093 Warsaw, Poland; Laboratory of Imaging Tissue Structure and Function, Nencki Institute of Experimental Biology, 3 Pasteur St., 02-093 Warsaw, Poland; Institute of Cell Biology, National Academy of Sciences of Ukraine, 14/16 Drahomanov St., Lviv, 79005 Ukraine; Randall Division of Cell and Molecular Biophysics and Cardiovascular Division, King’s College London, Guy’s Campus, London, SE1 1UL UK

**Keywords:** Actin cytoskeleton, Adhesion, Cardiomyocytes, Cell migration, Differentiation, Endoplasmic reticulum, Golgi apparatus, Neuromuscular junction, Talin

## Abstract

The important role of unconventional myosin VI (MVI) in skeletal and cardiac muscle has been recently postulated (Karolczak et al. in Histochem Cell Biol 139:873–885, 2013). Here, we addressed for the first time a role for this unique myosin motor in myogenic cells as well as during their differentiation into myotubes. During myoblast differentiation, the isoform expression pattern of MVI and its subcellular localization underwent changes. In undifferentiated myoblasts, MVI-stained puncti were seen throughout the cytoplasm and were in close proximity to actin filaments, Golgi apparatus, vinculin-, and talin-rich focal adhesion as well as endoplasmic reticulum. Colocalization of MVI with endoplasmic reticulum was enhanced during myotube formation, and differentiation-dependent association was also seen in sarcoplasmic reticulum of neonatal rat cardiomyocytes (NRCs). Moreover, we observed enrichment of MVI in myotube regions containing acetylcholine receptor-rich clusters, suggesting its involvement in the organization of the muscle postsynaptic machinery. Overexpression of the H246R MVI mutant (associated with hypertrophic cardiomyopathy) in myoblasts and NRCs caused the formation of abnormally large intracellular vesicles. MVI knockdown caused changes in myoblast morphology and inhibition of their migration. On the subcellular level, MVI-depleted myoblasts exhibited aberrations in the organization of actin cytoskeleton and adhesive structures as well as in integrity of Golgi apparatus and endoplasmic reticulum. Also, MVI depletion or overexpression of H246R mutant caused the formation of significantly wider or aberrant myotubes, respectively, indicative of involvement of MVI in myoblast differentiation. The presented results suggest an important role for MVI in myogenic cells and possibly in myoblast differentiation.

## Introduction

Myosins form a structurally and functionally diverse superfamily of actin-based molecular motors expressed in all eukaryotic cells that consists of more than 35 distinct families (Odronitz and Kollmar 2007). Besides the nomenclature based on family affiliation, myosins are also termed as conventional (i.e., belonging to class II muscle and non-muscle myosins able to form filaments) or unconventional (all the remaining families).

So far, myosin I isoforms, IA, IB, and IC; myosin VA; myosin VIIA; myosins IXA and IXB as well as myosins XVIIIA and B were identified in striated muscle and/or in myogenic cells, and shown to be potentially involved in differentiation of myoblasts into myotubes, and in muscle development and/or regeneration (Wells et al. 1997; Salamon et al. 2003; Redowicz 2007; Roder et al. 2012; Cao et al. 2014; Sun et al. 2014). Specifically, myosin XVIIIB plays a key role in cardiac muscle development and is involved in Drosophila myoblast fusion, and PDZ-containing myosin XVIIIA isoform has been recently found in zebrafish to maintain the stable attachment of myofibers to extracellular matrix and muscle integrity during early development (Ajima et al. 2008; Bonn et al. 2013; Cao et al. 2014).

Recently, we have shown that myosin VI (MVI) is expressed in striated muscles, where it seems to be involved in the function of the sarcoplasmic reticulum and neuromuscular junction, and possibly in gene transcription (Karolczak et al. 2013, 2014). Notably, its amount was significantly increased in atrophic fibers of muscle biopsies obtained from patients with several myopathies (Karolczak et al. 2014). In humans, a point mutation (H246R) within the MVI gene was associated with cardiac hypertrophy, suggesting an important role of this molecular motor in striated muscles (Mohiddin et al. 2004).

**Fig. 1:**
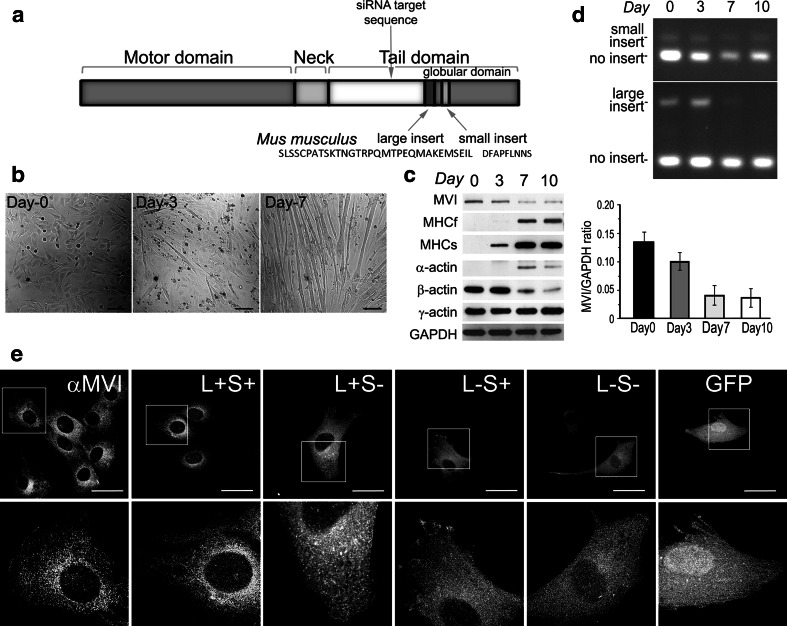
MVI expression during differentiation. **a** Diagram representing the domain structure of MVI. Location of large and small inserts of the mouse MVI is indicated. **b** Phase-contrast images of C2C12 myotubes after 3 (day-3) and 7 (day-7) days of differentiation. Day-0 myoblasts shortly before switching to 2 % horse serum-containing medium. **c** Analysis of MVI, fast (MHCf) and slow (MHCs) myosin heavy chains as well as α-, β-, and γ-actin levels by immunoblot at indicated days after the initiation of differentiation. A representative immunoblot is shown. *Right panel*: a quantitative analysis of the MVI content during myoblast differentiation with respect to the level of glyceraldehyde-3-phosphate dehydrogenase (GAPDH). Values are means ± SD; ***p < 0.001, as measured by Student’s *t* test. **d** Assessment of MVI splice variant levels by RT-PCR in differentiating myoblasts. The products obtained with primers designed to produce fragments containing either small or large inserts, as indicated in the figure. **e** MVI and its splice variants distribution in undifferentiated myoblasts. The endogenous MVI localization was assessed with anti-porcine MVI antibody (αMVI). Myoblasts were also transfected with GFP-tagged human MVI constructs encoding MVI variants with: both inserts (L+S+), the large insert (L+S−), the small insert (L−S+), and without inserts (L−S−). A plasmid encoding GFP alone was used as control. *Lower panels* ×~3 magnification of the areas marked in the corresponding *upper panels*. *Bars* in (**b**, **e**), 100 and 20 μm, respectively

The MVI heavy chain (MW ~140 kDa) has a domain pattern characteristic for all the myosins. It has (i) an N-terminal motor domain (with the actin- and ATP-binding sites); (ii) a neck, which binds two calmodulin molecules; and (iii) a tail domain, with C-terminal part forming a globular domain involved in cargo binding and/or interaction with its partners (Fig. 1a; Tumbarello et al. 2013). Four splice variants of MVI can be formed in mammalian cells due to the presence of two inserts (small and large) within the tail domain (Fig. 1a; Aschenbrenner et al. 2003; Au et al. 2007). These authors postulate that the inserts could determine MVI subcellular localization and thus function.

MVI functions through its interaction with actin (via the N-terminal motor domain) and partner proteins (via the C-terminal cargo domain). Two tail regions were found to be involved in binding partner recognition: a positively charged RRL region and a hydrophobic WWY region (Tumbarello et al. 2013). Also, a positively charged cluster of the MVI C-terminal globular tail was shown to bind to PIP_2_-containing liposomes, possibly aiding in the binding partners recognition (Spudich et al. 2007). It has been recently shown that MVI must dimerize and deploy its unusual lever arm in order to perform its cellular functions (Mukherjea et al. 2014).

Numerous tissue- and cell-specific MVI-binding partners have been already identified in mammals; among them are adaptor proteins, enzymes, and proteins involved in the regulation of cytoskeleton dynamics (Tumbarello et al. 2013; Majewski et al. 2012). We have recently shown that in skeletal muscle, MVI seems to interact with TOM1 (target of myb1 homolog isoform 1), a protein involved in intracellular transport and autophagy, FMRP (fragile X mental retardation protein involved in mRNA transport) as well as with hnRNP proteins, heterogeneous ribonucleoproteins involved in the RNA transport and maturation (Karolczak et al. 2013).

Unlike other known myosins, MVI moves backward (i.e., toward the minus, pointed end of actin filaments), implying that it has a role distinct from other myosins (Wells et al. 1999). It has been reported that MVI is involved in endocytosis and intracellular transport of vesicles and organelles, cell migration, maintenance of Golgi apparatus, actin cytoskeleton organization, and possibly in gene transcription (Jung et al. 2006; Vreugde et al. 2006; Sweeney and Houdusse 2007, 2010; Chibalina et al. 2009; Majewski et al. 2011).

Although unconventional myosins could be involved in muscle precursor function (Redowicz 2007), no studies have been published to date on the role of MVI in myogenic cells and their differentiation. Here, we present for the first time the data, indicating that in myogenic cells, MVI plays an important role in myoblast function and their differentiation into the myotube by regulating the organization of the actin cytoskeleton, maintenance of endoplasmic reticulum and Golgi apparatus, and the formation of cell adhesions and muscle postsynaptic machinery.

## Materials and methods

### Cell culture

C2C12 mouse myoblasts (American Type Culture Collection, USA), kindly provided by Prof. Krzysztof Zablocki from the Nencki Institute, were maintained in DMEM containing 4.5 g/l glucose and supplemented with 10 % heat-inactivated fetal bovine serum (FBS), antibiotics (1000 UI/ml penicillin and 1000 UI/ml), and 4 mM l-glutamine at 37 °C in humidified air containing 5 % CO_2_. Differentiation was initiated upon reaching confluence (considered as day 0) by transferring to medium containing 2 % horse serum (HS) instead of 10 % FBS, and the culture was continued for up to next 7–10 days. To observe postsynaptic structures, cells were differentiated in 8-well Permanox chamber slides (Sigma-Aldrich, USA) coated with laminin (Invitrogen, USA) as described by Proszynski et al. (2009). The differentiation was monitored with the Nikon inverted microscope equipped with a Nikon PLAN 10DIC/0.25 long-working-distance objective. The images were collected using cooled CCD camera (Retiga 1300, QImaging Co., Canada),

### Isolation and culture of neonatal rat cardiomyocytes

Newborn rat hearts were dissected and digested using the Neonatal Cardiomyocyte Isolation System from Worthington (Worthington Biochemical Corp., USA) and cultured up to 8 days as described previously (Iskratsch et al. 2010). Housing and killing procedures were performed in compliance with the European Communities Council Directive of November 24, 1986 (86/609/EEC).

### Myosin VI splice variant analysis

RT-PCR was performed as described by Karolczak et al. (2013) using the following primers flanking the sequence of the small (forward: 5′-GCTCCCAAGTCGGTTACT-3′ and reverse: 5′-TTGTTCTGAGGGTCTTTGTA-3′) and large (forward 5′-GATGAGGCACAGGGTGAC-3′ and reverse 5′-GCGTATTTCCATTTACTGAGA-3′) inserts of the mouse myosin VI gene as well as glyceraldehyde-3-phosphate dehydrogenase (GAPDH) gene as an internal control. PCR products were run on 3 % agarose gel. The location of the inserts and their sequence is depicted in Fig. 1a.

### MVI overexpression

Plasmids encoding human MVI variants (gene ID Q9UM540), namely pEGFP-C3-MVI FL+S+ and pEGFP-C3-MVI FL−S+ encoding the small insert-containing variants with and without large insert, respectively, were generously provided by Dr. Tama Hasson from University of California, Los Angeles (Dance et al. 2004). Plasmids containing the variant without the small insert (pEGFP-C3-MVI FL+S−) and without any insert (pEGFP-C3-MVI FL−S−) were generated in our laboratory using the following primers: forward 5′-CCTCAGCAAAACCCAGCAGCTCAGATTCCT-3′ and reverse 5′-ATAATCAGTAACAGACTTTGGAGCACGTTG-3′. Plasmid pEGFP-C3-MVI FL+S+H/R encoding MVI with point mutation (His246Arg) was obtained using forward 5′-AAGAAATTATAGGATCTTTTATAGGTTGTGT-3′ and reverse 5′-ACACAACCTATAAAAGATCCTATAATTTCTT-3′ primers. C2C12 cells (untreated or treated with MVI knockdown) were next plated on coverslips in 35-mm dishes, cultured for 24 h, and transfected with the plasmids using Lipofectamine 2000 (Invitrogen, USA) according to the manufacturer’s protocol. Plasmid pEGP-C3 was used as control.

### siRNA knockdown of MVI

C2C12 cell line with MVI stable knockdown (MVI-KD cell line) was generated based on pSilencer 2.1-U6 hygro vector system (Ambion Inc., USA) essentially as described by Majewski et al. (2011). 5′-AACTACGCGATACAATCAATA-3′ siRNA sequence against a coding region of mouse MVI mRNA (gene ID Q64331) was used with the corresponding scrambled sequence as a negative control (5′-ATAACATACCGTACGAATAAC-3′). Of note, the chosen shRNA targeted the different MVI region than the ones used for the protein depletion in PC12 cells (Majewski et al. 2011). C2C12 cells were transfected using Lipofectamine 2000 and then selected on hygromycin B (Sigma-Aldrich, USA). C2C12 cells were also transiently transfected with the pSuper vector containing siRNA sequence against a coding region of rat MVI (gene ID D4A5I9) mRNA (5′-CTTCGCGATACAATCAACA-3′, 3395–3413—partially overlapping with the sequence used for obtaining the stable MVI knockdown cells) and pSuper vector with a scrambled MVI sequence as control on day 1 (i.e., 1 day after transferring to the differentiating conditions), and then, cells were differentiated for next 6 days. The differentiation was monitored as described above. MVI expression level was assessed by the Western blot as well as RT-PCR.

### Antibodies and fluorescent markers

Rabbit polyclonal antibody directed against the amino acid residues 1049–1054 of the porcine myosin VI heavy chain was purchased from Proteus (USA), and anti-β-actin was commercially generated for us by EZBiolab (USA). Goat polyclonal antibody against human calreticulin was a gift from Dr. Marek Michalak from the University of Alberta (Canada). Sheep polyclonal antibody against γ-actin was from Merck Millipore (USA). The following monoclonal antibodies were also used: anti-α-actinin and anti-β-actin from Sigma-Aldrich (Germany), anti-α-actin and anti-GRP78 from Abcam (UK), anti-GAPDH (glyceraldehyde 3-phosphodehydrogenase) and anti-SERCA 2 from Merck Millipore (USA), anti-GM130 from BD Biosciences (USA), as well as anti-fast and slow myosin heavy chains were from Abcam, anti-talin was from Santa Cruz Biotechnology (USA), and anti-vinculin from Sigma-Aldrich (USA). TO-PRO^®^-3 iodide staining chromatin, Alexa Flour 488-conjugated bungarotoxin staining muscle synapses, and Alexa Flour 546(or 488)-conjugated phalloidin were purchased from Invitrogen (USA).

### Immunoblotting

Cells were lysed in an ice-cold buffer containing 50 mM Tris–HCl, pH 7.5; 150 mM NaCl; 0.1 % Triton X-100; 1 mM DTT; 1 mM EGTA; 1 mM PMSF; and complete protease inhibitor cocktail (Roche). Cell lysates (10–20 μg of protein per well) were separated using 10 or 12 % SDS–PAGE gels and then transferred to a nitrocellulose membrane. Western blot was performed as described by Karolczak et al. (2013), and the bands were detected by the enhanced chemiluminescence technique (ECL) based on the activity of horse radish peroxidase conjugated with secondary antibodies. The blots were subjected to densitometric analysis, and Student’s *t* test was used to evaluate the quantitative data from at least three analyses of the lysates from independent experiments.

### Immunolocalization studies

Cells on coverslips were fixed in 4 % formaldehyde in phosphate-buffered saline, pH 7.4 (PBS), for 20 min at room temperature, washed with PBS, blocked in 2 % horse serum, and permeabilized with 0.02 % Triton X-100 in PBS for 30 min at room temperature. Coverslips were then incubated for 2 h at room temperature or overnight at 4 °C with anti-MVI rabbit polyclonal antibody diluted 1:50 and cell compartment markers: sarcoplasmic reticulum: anti-calreticulin (1:50), anti-GRP78 (1:500), and anti-SERCA2 antibodies (1:50); Golgi apparatus: anti-GM130 (1:100); adhesive structures: anti-vinculin (1:50) and anti-talin (1:50); or the cytoskeleton: anti-α-actinin, anti-γ-actin, or anti-β-actin (all at 1:1000 dilution), washed with PBS, followed by incubation with 1 µg/ml Alexa Fluor 488-conjugated goat anti-rabbit IgG or Alexa Fluor 546-conjugated goat anti-mouse, donkey anti-goat, or donkey anti-sheep IgG (Molecular Probes, Invitrogen). Vectashield mounting medium (Vector Laboratories, USA) was used to mount the slides. All fluorescence images were acquired on Leica TCS SP5 o SP8 confocal laser scanning microscope equipped with a HCX PL APO 40×/1.25–0.75 Oil Cs or HCX PL APO 63/1.4 Oil objective. An argon laser at 488 nm, a diode-pumped solid-state laser 561 nm, and a helium neon laser at 594 nm were used to excite Alexa Flour 488, 555, and 546 fluorescence, respectively. Optical sections (1024 pixels × 1024 pixels × 12 bits/pixel) were collected usually at 0.30 µm *z*-spacing. In double or triple immunostaining, special care was taken to control for any possible cross talk of the detection systems. The spectral ranges of the detectors were carefully adjusted for the detectors, and the different channels were always scanned sequentially. For negative controls, the primary antibody was omitted. Confocal sequences were further processed by lens point-spread function deconvolution using Huygens Professional 64 version 14.06 (Scientific Volume Imaging BV, the Netherlands) software. Point-spread functions were calculated according to profiles of known Leica lens parameters, not measured. Synthetic imaging (Z stack reconstructions and multichannel image creation) was performed in the Fiji distribution of ImageJ software.

### Analysis of cell shape

The analysis of shape of the examined cells was performed in the ImageJ software using the edge detection function based on the cell outlines. Circularity (defined as: 4π × area/perimeter^2^) and roundness (defined as aspect ratio of the minor/major axis) parameters were calculated; for both, 1.0 indicated a perfect circle, and smaller values indicated oblong and non-circular objects. The analyses were done for at least 100 cells of each examined condition for at least two independent experiments.

### Cell compartment analysis

MetaMorph software (Molecular Devices, USA, distributed as Leica MM AF version 1.4.0) was used for the quantification studies. Golgi apparatus and endoplasmic reticulum areas were calculated after outlining their surface based on the staining for GM130 and calreticulin, respectively. The entire cell area was assessed after outlining the cell contours based on the Alexa Flour 546-phalloidin staining. At least 80 images of cell centers from each experimental condition from two independent experiments were analyzed. Assessment of the size of focal adhesions was based on the number of vinculin-stained pixels and performed in at least 20 cells from each experimental condition from two independent experiments. Statistical analysis was performed using Student’s *t* test.

### Proximity ligation assay (PLA)

The assay was performed according to the manufacturer’s instructions (Olink Bioscience, Sweden). Briefly, myoblasts and mature myotubes were blocked after fixation in Duolink blocking solution in a humidity chamber for 30 min at 37 °C and incubated with primary antibodies: polyclonal anti-MVI (1:50) and anti-talin (1:50) in Duolink antibody diluent solution for 3 h at 37 °C. Cells were next washed twice in a wash buffer for 5 min at room temperature. For secondary antibodies conjugated with oligonucleotides, PLA probe anti-mouse MINUS and PLA probe anti-rabbit PLUS were applied in Duolink antibody diluent solution for 1 h at 37 °C and washed twice in a wash buffer for 5 min. Duolink assay was further performed strictly according to the manufacturer’s instructions. Duolink red fluorescence detection kit was used with excitation at 594 nm and emission at 624 nm. For negative controls, the primary antibodies were omitted.

### Random motility assay

To perform migration assays, 12,500 of untreated C2C12, scrambled or MVI-KD cells were seeded into 24-well cell culture plate and grown for 24 h in an appropriate culture medium. Then, three different areas per each cell type were photographed in DIC Nomarski contrast at 10× magnification every 10 min for 16 h using Retiga 1300 cooled CCD camera (QImaging Inc.) and Nikon Diaphot microscope equipped with an environmental chamber. At least 30 cells of each experimental condition were tracked using MetaMorph software. Cell movement between frames was assessed by the analysis of the image template shift to the position of highest similarity in the following frame. The similarity was measured by the cross-correlation function value. Statistical analysis was performed using Student’s *t* test.

## Results

### Myosin VI expression in C2C12 myoblasts

MVI is expressed in undifferentiated (day-0) as well as in differentiating (day-3, day-7, and day-10) myoblasts (Fig. 1b, c), and its synthesis gradually decreases during differentiation (Fig. 1c). In contrast, levels of skeletal muscle myosin heavy chains (fast and slow isoforms) and skeletal muscle α-actin increase (Fig. 1c), as expected (Scordilis et al. 1981; Lin and Lin 1986; Hayward et al. 1988). Consistently with previous observations, we also observed a decrease in β-actin expression (Lin and Lin 1986; Hayward et al. 1988), but we did not notice any substantial difference in γ-actin expression (Fig. 1c).

Four MVI posttranscriptional splice variants (Fig. 1a) can be formed in mammals due to the presence of two inserts, large and small, in its C-terminal globular tail domain (Aschenbrenner et al. 2003; Au et al. 2007). We made an attempt to assess which isoforms are expressed in murine myoblasts (Fig. 1d). In undifferentiated myoblasts (day-0), the isoforms both with inserts and without the inserts are expressed. This pattern changes during differentiation, and in day-10 myotubes, the variants without the inserts were the predominant ones.

To examine MVI expression and its cellular distribution, we performed immunofluorescence staining of undifferentiated myoblasts (Fig. 1e, αMVI). Endogenous MVI localized to puncti distributed within the entire cytoplasm, including the leading edge and the perinuclear area. A similar observation was made for overexpressed GFP-tagged MVI constructs encoding all four splice variants with the exception of the variant with both inserts (L+S+), which had a higher tendency to localize to the perinuclear area (Fig. 1e).

### MVI distribution

**Fig. 2:**
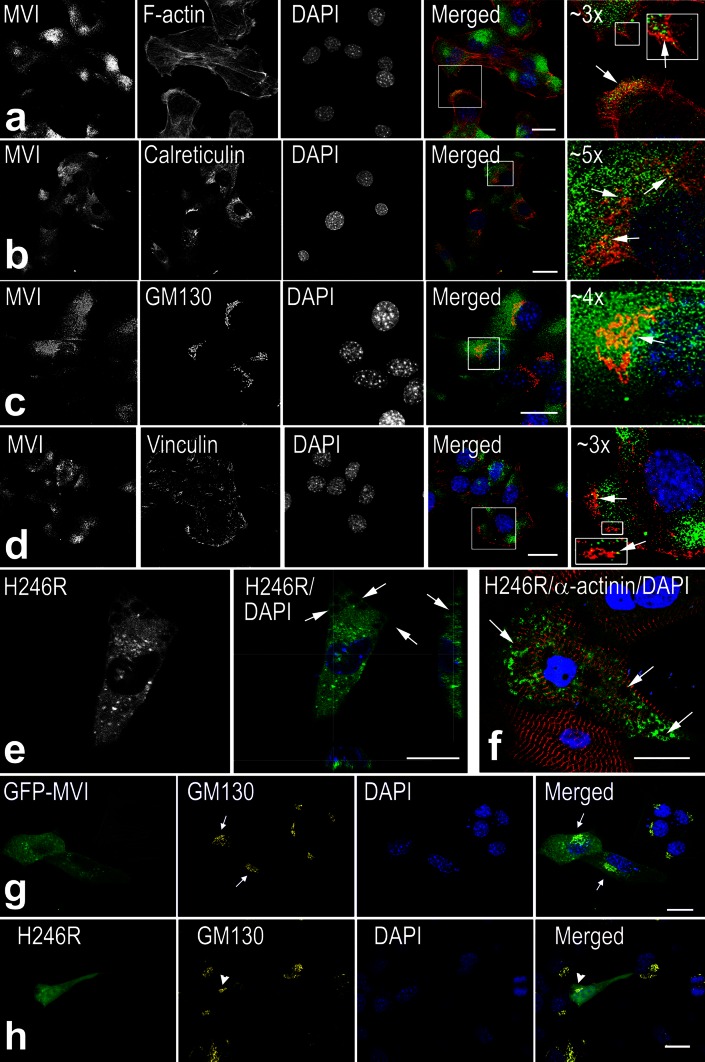
MVI localization to different compartments of undifferentiated myoblasts. MVI is visualized in *green* with anti-MVI antibody (**a**–**d**) or as GFP-associated fluorescence (**e**–**h**), and nuclei were stained with DAPI (**a**–**h**). **a** MVI is present in the regions next to cortical actin (in *red*, stained with Alexa Flour 536-conjugated phalloidin). **b**, **c** MVI is in the close proximity to calreticulin (in *red*), an endoplasmic reticulum marker, and to GM130 (in *red*), **a** Golgi apparatus marker, respectively. **d** MVI is present next to vinculin-containing (in *red*) adhesive structures. Regions indicated in the merged images are shown at higher magnification in the *right panel*. **e** Overexpression of H246R MVI mutant (in *green*) myoblasts with MVI knockdown; *right panel*, merged with DAPI staining. **f** Overexpression of H246R MVI mutant (in *green*) in 2-day rat neonatal cardiomyocytes. In *red*, staining for α-actinin, the marker of *Z* lines. *Arrows* on e and f indicate vacuole-like structures. **g**, **h**, Day-0 myoblasts overexpressing the GFP-fused wild-type MVI (GFP-MVI) or H246R mutant, respectively. Golgi cisternae (in *yellow*) were stained with anti-GM130 monoclonal antibody. *Arrows* in (**g**), Golgi cisternae in cells overexpressing GFP-MVI, and *arrowhead* in (**h**), Golgi cisternae in the cell overexpressing the MVI mutant. *Bars* 20 μm

**Fig. 3:**
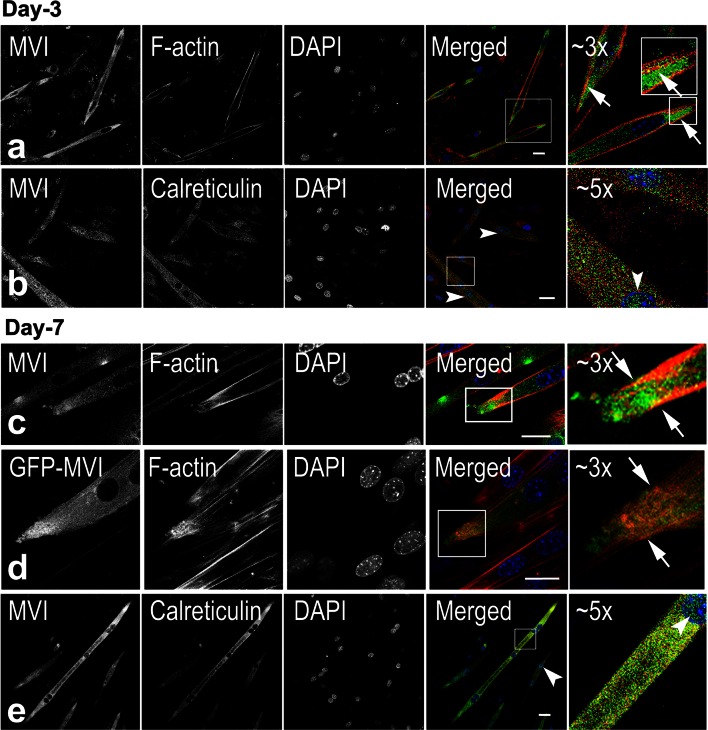
MVI localization during myoblast differentiation. MVI in day-3 (**a**, **b**) and day-7 (**c**, **e**) cells is visualized in *green* with anti-MVI antibody, and in d as GFP-associated fluorescence. In a as well as in (**c**, **d**), MVI localizes to actin filaments, especially at myotube edges. **b**, **e**, MVI is in close proximity to calreticulin (in *red*), especially in day-7 myotubes. **d** Myotube overexpressing GFP-tagged full-length MVI (GFP-MVI). GFP-MVI is also enriched at the actin-rich myotube edge. The *far* *right panels*, magnification (as marked on the figure) of the regions indicated in the *merged panels*. *Arrows* point to colocalization of MVI with the examined markers and *arrowheads* to the nuclear MVI presence. *Bars* 20 μm

To define MVI distribution in undifferentiated and differentiating myoblasts, stainings for MVI and markers of various cellular compartments were performed (Figs. 2, 3).

In undifferentiated myoblasts, MVI was ubiquitously distributed within the cytoplasm, also in areas intensely stained for actin filaments (Fig. 2a).

In undifferentiated myoblasts, MVI was also seen next to endoplasmic reticulum (ER) and Golgi apparatus cisternae, as visualized by staining with anti-calreticulin and anti-GM130 antibodies, respectively (Fig. 2b, c). MVI was also seen in the regions adjacent to adhesive structures stained with anti-vinculin antibody (Fig. 2d).

To further explore the potential MVI function in myoblasts, we transfected the MVI knockdown cells with a construct encoding the human H246R MVI mutant (Fig. 2e). This point mutation located within the motor domain was previously found to be associated with hypertrophic cardiomyopathy and predicted to inactivate MVI motor functions (Mohiddin et al. 2004); therefore, we used it as a loss-of-function MVI form with defective motor activity. Our preliminary data indicate, however, that ability of the mutant to bind to filamentous actin is not impaired (not shown). Expression of the mutant protein caused the formation of very large vesicles (with a diameter of about 3–5 μm) that localized at the cell periphery (Fig. 2e, arrows). These vacuole-like vesicles resembled those found in a patient with mutation in dynamin-2 gene (Karolczak et al. 2014), indicative of impairment of endocytic vesicle processing. Vesicles with similar diameter were also observed in rat neonatal cardiomyocytes (Fig. 2f) upon expression of MVI mutant protein. Also, overexpression of C2C12 cells with the H246R MVI mutant fused with GFP (Fig. 2h) affected Golgi apparatus organization as the cisternae stained with anti-GM130 antibody were more compact (marked by arrowhead) that the ones visible both in untreated cells or cells overexpressing wild-type MVI (GFP-MVI, Fig. 2g, marked with arrows).

A similar localization pattern of MVI was observed for day-3 elongated myoblasts (Fig. 3a, b) and for day-7 multinuclear myotubes (Fig. 3c–e). MVI was distributed throughout the cytoplasm but concentrated at the cell edges (Fig. 3a, c, arrows). The concentration of MVI at the tips of myotubes was independently confirmed in myotubes overexpressing GFP-MVI construct (Fig. 3d). As for myoblasts, MVI was also observed in close proximity to the ER (Fig. 3b, e, arrows), and its association with this compartment was more prominent in day-7 myotubes. Occasionally, as for muscle (Karolczak et al. 2013), MVI was also detected in the nuclei (Fig. 3b, f, arrowheads). Colocalization of MVI with the Golgi marker in differentiating myoblasts and myotubes was not so evident as for undifferentiated cells (not shown).

**Fig. 4:**
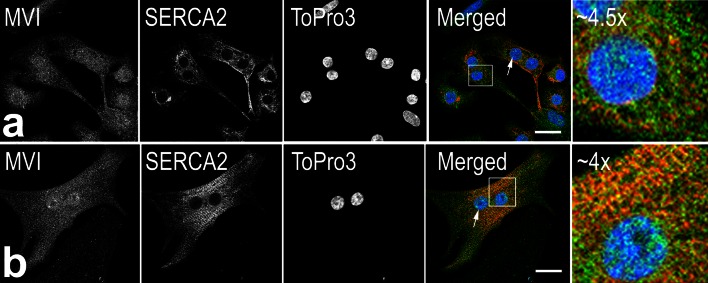
MVI colocalization with sarcoplasmic reticulum in neonatal rat cardiomyocytes.** a** and** b**, Localization of MVI (in *green*) and SERCA2 (in *red*) in day-2 and day-8 cardiomyocytes, respectively. *Arrows* point to the nuclear MVI presence. The *far right panels*, magnification (as marked in the figure) of the regions indicated in the *merged panels*. Nuclei were stained with TO-PRO^®^-3 (in *blue*). *Bars* 10 μm

Differentiation-dependent increase in colocalization of MVI with SERCA-2, the marker of the cardiac ER, was also observed for neonatal rat cardiomyocytes (NRC; Fig. 4). The two proteins colocalized more clearly in the cells cultured for 8 days (Fig. 4b) than in those cultured for 2 days (Fig. 4a) after isolation from the heart. MVI was also present in the cardiomyocyte nuclei (Fig. 4, arrows).

### MVI is enriched in myotube regions stained with α-bungarotoxin

We have previously shown that in skeletal muscle, MVI was present in the postsynaptic region of the neuromuscular junction (NMJ) in an innervation-dependent manner, indicating its possible involvement in the muscle synapse function/maintenance (Karolczak et al. 2013). To test whether MVI could be also involved in the development of the NMJ, we stained C2C12 myoblast-derived myotubes cultured on a laminin-coated surface with fluorescently-labeled α-bungarotoxin (BTX), which binds to acetylcholine receptors (AChRs), components of the postsynaptic machinery at the NMJ. Myotubes cultured under these conditions were previously shown to form AChR-rich structures resembling the postsynaptic moiety of the neuromuscular junction (Proszynski et al. 2009; Kummer et al. 2004; Proszynski and Sanes 2013).

**Fig. 5:**
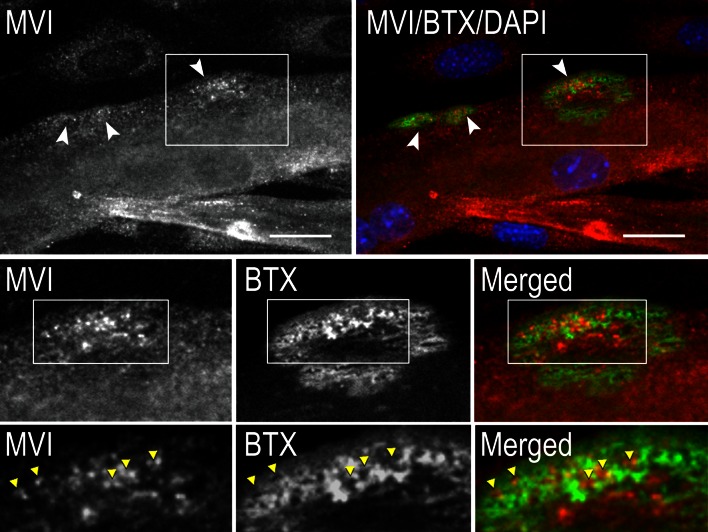
MVI is present within acetylcholine receptor-rich clusters. **a** *upper panels*, MVI (in *red*) is enriched in postsynaptic clusters (in *green*, BTX). In *blue*, the myotube nuclei stained with DAPI. Middle panels, ×~2 magnification of regions marked in *upper panels* and *bottom panels*, ×~1.5 magnification of regions marked in the *middle panels*. *Arrowheads* point to the regions stained for MVI but lacking the BTX-staining. *Bars* 20 μm

The majority of myotubes developed AChR-rich structures, which varied in size and organization status (Fig. 5). We observed the enrichment of the MVI presence in the regions corresponding to the nascent AChR-rich clusters (Fig. 5, arrowheads in upper panels). Notably, although MVI was present within the clusters, it did not colocalize with the AChR, but was concentrated in AChR-poor regions surrounded by or adjacent to the receptors (Fig. 5, lower panels, yellow arrowheads).

### Effect of MVI knockdown on cell morphology and actin cytoskeleton organization

**Fig. 6:**
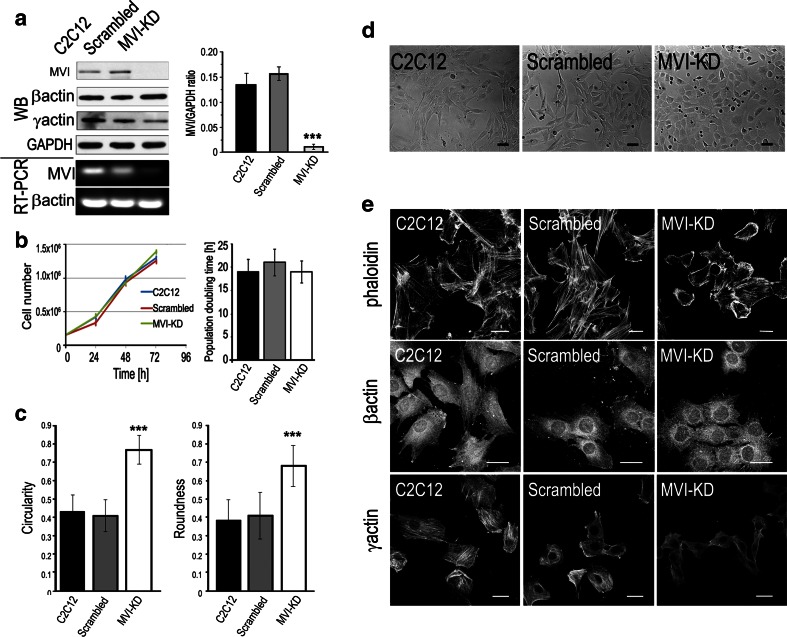
MVI depletion affects cell morphology and actin cytoskeleton organization. **a** Immunoblot analysis (WB) of lysates of untransfected myoblasts (C2C12) and myoblasts transfected with a scrambled or MVI shRNA sequence (MVI-KD), probed with anti-MVI and anti-β- and γ-actin isoforms. *Bottom panels* RT-PCR analysis of the expression of MVI and β-actin in the same cell lines. *Right panel* a quantitative analysis of the MVI content with respect to the GAPDH level. **b** Number of cells per well in each cell line was counted after 24, 48, and 72 h. The population doubling time for each culture was found not to be statistically significantly different. The values presented as means ± SD were obtained from three independent experiments. **c** Assessment of circularity and roundness parameters of the examined cell lines. The analysis was performed for about 100 cell for each cell line from two independent experiments, **d** Phase-contrast images of untreated (C2C12), scrambled, and MVI-KD myoblasts. **e** Untreated, scrambled, and MVI-KD cells were stained for filamentous actin with Alexa Flour 647-conjugated phalloidin as well as with antibodies against γ- and β-actin isoforms. Values in **a**–**c** are means ± SD; ***p < 0.001, as measured by Student’s *t* test. *Bars* 20 μm

To elucidate the role of MVI in myoblasts (and possibly in their differentiation), we created a C2C12 cell line with stable expression of a MVI shRNA construct. The knockdown efficiency was confirmed by immunoblotting (WB) and RT-PCR (Fig. 6a). We tested three different shRNA sequences (not shown) and used the one that gave the strongest reduction in MVI expression for all subsequent experiments. It corresponded to bases 3403–3423 of the mouse *Myo6* sequence, within the MVI tail domain (see Fig. 1a). As controls, we used untransfected C2C12 cells and the cells transfected with the scrambled sequence. MVI knockdown did not significantly affect myoblast growth and proliferation (Fig. 6b), as compared to control cells.

Morphological analysis revealed that MVI knockdown cells (MVI-KD) were more oval in comparison with untransfected and scrambled myoblasts (Fig. 6c, d). Staining for filamentous actin with Alexa-phalloidin revealed that MVI-KD cells formed fewer stress fibers than control and scrambled cells, suggesting that MVI depletion evoked changes in the organization of the actin cytoskeleton (Fig. 6e). Since there is no significant difference in binding of phalloidin to microfilaments formed by the mammalian actin isoforms (Allen et al. 1996), we performed Western blot analysis (Fig. 6a) and immunostaining with antibodies specific for β- and γ-actin isoforms (Fig. 6e). It should be emphasized that the antibodies recognize both monomeric and filamentous actin. In MVI-KD cells (Fig. 6a), there was a slight increase in total β-actin content and a decrease in γ-actin content compared to untransfected and scrambled cells. Consistent with immunoblot results, fluorescence intensity of γ-actin staining was lower in MVI-KD cells (Fig. 6e).

### MVI knockdown affects Golgi and ER organization

**Fig. 7:**
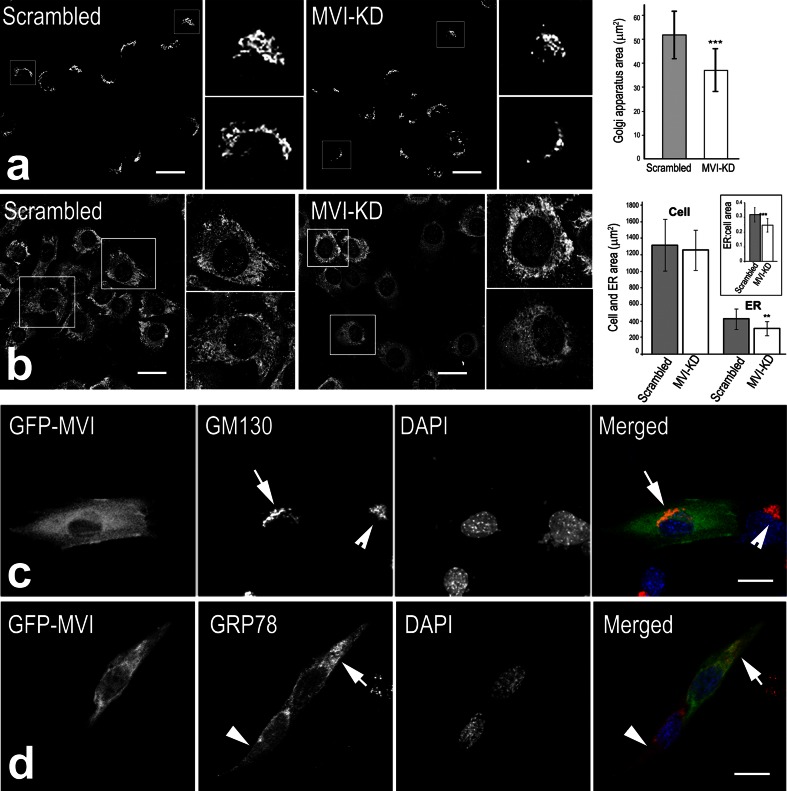
MVI depletion affects Golgi apparatus and endoplasmic reticulum. **a**, **b** Staining for GM130 and calreticulin in the scrambled and MVI-KD cells, respectively. The *smaller right panels* in (**a**, **b**) represent ×~2−4 magnification of the regions marked in l*arger panels*. *Right panel in (a),* assessment of the Golgi area in the scrambled and MVI-KD cells. *Right panels* in (**b**), assessment of the whole cell and the ER areas in the scrambled and MVI-KD cells. *Inset* quantification of the ratio of the area of ER to the area of the cell for each of the examined cells. The analyses were performed for at least 80 cells from each examined condition from two independent experiments. Values are means ± SD. ***p < 0.001; *p < 0.01 as measured by Student’s *t* test. **c** Overexpression of GFP-tagged human MVI (GFP-MVI, in *green*) in MVI-KD cell (*left cell*) restored to some extent morphology of the Golgi apparatus (stained in *red* for GM130, marked by *arrow*). The Golgi apparatus in MVI-KD untransfected cell (on the *right*, marked by *arrowhead*) remains to be compact. **d** Overexpression of GFP-tagged human MVI (GFP-MVI, in *green*) affects the ER (stained for GRB78, in *red*) organization of the transfected (an *arrow*) but not of untransfected (an *arrowhead*) MVI-KD cells. Nuclei are stained with DAPI (in *blue*). *Bars* in (**a**–**d**), 20 μm

Since the involvement of MVI in Golgi apparatus maintenance was previously shown for fibroblasts and PC12 neurosecretory cells (Warner et al. 2003; Majewski et al. 2011), we checked whether Golgi apparatus organization was also impaired in MVI-KD myoblasts. As expected, Golgi morphology in MVI-KD cells was affected: the cisternae were fragmented, and their area was reduced by about 27 % in comparison with scrambled cells (Fig. 7a).

Because MVI was also found next to the ER, especially in differentiating cells, we checked whether MVI knockdown could also affect morphology of this compartment. In MVI-KD cells, the ER appeared to be more compact and less branched in comparison with scrambled cells (Fig. 7b). Moreover, the area covered by the ER in MVI-KD cells was about 25 % smaller than in scrambled cells (Fig. 7b).

To assess whether these changes were induced by depletion of MVI, we transfected MVI-KD cells with GFP-tagged human MVI (GFP-MVI) to rescue the phenotype and then stained them with either the Golgi (Fig. 7c) or ER (Fig. 7d) marker. Ectopic expression of MVI rescued to some extent the phenotype observed in MVI-KD cells. Both the Golgi cisternae and the ER in GFP-MVI-expressing cells (marked by arrows) more resembled these in scrambled cells than those in non-rescued MVI-KD cells.

### MVI knockdown affects adhesion complex formation

**Fig. 8:**
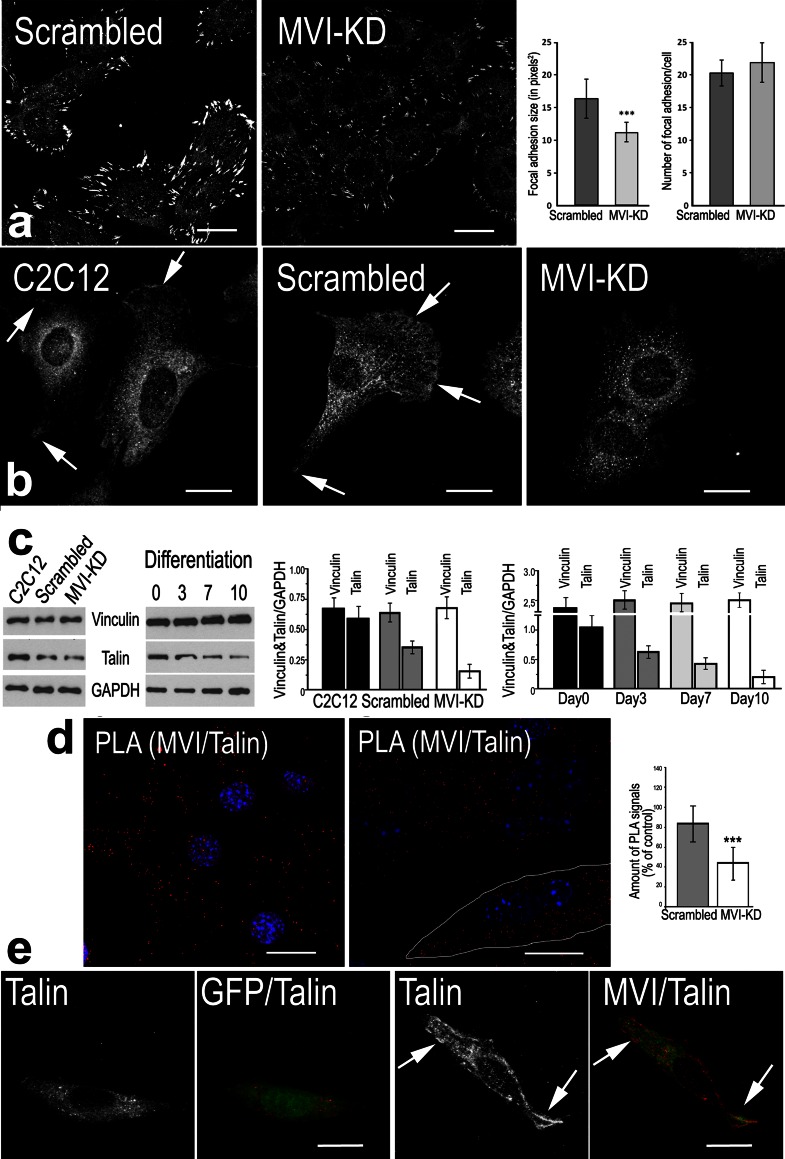
MVI in adhesive structures. **a** Anti-vinculin staining in the scrambled and MVI-KD myoblasts. *Graphs* show the quantification of the size (on the *left*, in pixels) and number (on the *right*) of vinculin-stained focal adhesions. **b** Anti-talin staining in untransfected (C2C12), scrambled, and MVI-KD myoblasts. *Arrows* point to the talin-positive cell edges. **c** Representative immunoblot of lysates of untreated, scrambled, and MVI-KD myoblasts (on the *left*) as well as of untreated myoblasts transferred to differentiating conditions for up to 10 days (on the *right*) probed with anti-vinculin, anti-talin, and anti-GAPDH antibodies. Graphs present quantitative analyses of vinculin and talin content relative to GAPDH in C2C12, scrambled and MVI-KD myoblasts as well as during differentiation (days 0–10); **d** PLA staining (in *red*) shows proximity of MVI and talin in untreated day-0 myoblasts (*left panel*) and day-10 myotubes (*middle panel* a myotube is contoured in *white*). In *blue*, nuclei stained with DAPI. Graph (*right panel*) presents the quantification of the total number of PLA signals in the scrambled and MVI-KD cells expressed as percent of control C2C12 myoblasts. At least 20 cells were quantified for each condition. The values in **a**–**c** are means ± SD. ***p < 0.001. **e** Effects of overexpression of GFP (*green*, *left panels*) and GFP-tagged human MVI (*green*, *right panels*) in MVI-KD cells on localization of talin (in *white* or *red*). Overexpression of GFP-MVI but not of GFP restored a wild-type-like talin staining pattern (*arrows*). *Bars* 20 μm

Changes in actin cytoskeleton organization, especially of the cortical region, indicate that MVI depletion could also affect cell adhesion. To address this problem, we immunostained MVI-KD and control (scrambled) cells with anti-vinculin and anti-talin antibodies (Fig. 8a, b, respectively). In MVI-KD cells, the vinculin-containing structures were smaller, and vinculin immunofluorescence intensity was lower (Fig. 8a). Quantitative analysis of vinculin-associated pixels confirmed a decrease in the size of focal adhesions in MVI-KD cells. Interestingly, the total number of the vinculin-containing adhesive structures was similar in MVI-KD and control cells (Fig. 8a).

Talin subcellular distribution was also affected in MVI-depleted cells. In untreated C2C12 myoblasts and cells transfected with scrambled shRNA, talin immunoreactivity was detected in the periphery of cells (Fig. 8b, arrows), but such localization was absent in MVI-KD cells (Fig. 8b). Importantly, the total expression level of talin but not of vinculin was profoundly affected by MVI depletion, as revealed by Western blot analysis (Fig. 8c, left panel and left graph). Moreover, the analysis of talin and vinculin expression during myoblast differentiation revealed a substantial decrease in talin but not of vinculin (Fig. 8c, right panel and right graph). Finally, talin and MVI are in close proximity to each other, as assessed by the PLA assay (Söderberg et al. 2006) performed in C2C12 myoblasts (Fig. 8d). This potential interaction is MVI-specific as the number of PLA signals was decreased to about 40 % in MVI-depleted myoblasts (Fig. 8d, graph). Also, overexpression of GFP-MVI and not of GFP alone in MVI-KD cells restored the peripheral talin distribution (Fig. 8e, right panels, arrows). Moreover, close proximity of talin and MVI was maintained also within the mature myotube (Fig. 8d, right image). These observations are in line with our data obtained by mass spectrometry in which we identified talin as a potential MVI-binding partner (Karolczak et al. in press). In that study, talin was efficiently precipitated with MVI globular tail domain from C2C12 cells extract at the day-0, day-3, and day-7 post-differentiation. These findings further imply functional interaction of MVI and talin in adhesive structure formation and organization.

### MVI knockdown impairs myoblast migration

**Fig. 9:**
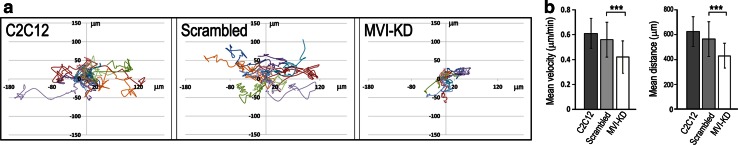
MVI involvement in cell migration. **a** Migration tracks (reoriented to zero in migration traces) of 10 randomly chosen nonproliferating untreated (C2C12), scrambled, or MVI-KD myoblasts. The values on *x-* and *y*-axes are given in μm. **b** Cell migration rate and mean distance were measured based on the analysis of tracks of each 30 untreated C2C12, scrambled, and MVI-KD cells. Values are means ± SD. ***p < 0.001

The observed changes in the cytoskeleton organization and adhesive structure formation in MVI-KD cells suggest that MVI depletion could also affect myoblast migration. Therefore, we assessed myoblast migration by the analysis of random migration by time-lapse microscopy without external chemotactic stimuli (Fig. 9). We tracked individual cells and measured their migration velocity and mean distance. MVI-KD moved in a less coordinated manner, i.e., they did not continue to move in one direction for a longer period of time (Fig. 9a). Moreover, MVI-KD cells migrated approximately 25 % slower than cells expressing scrambled shRNA and approximately 30 % slower than untransfected cells (Fig. 9b). Also, MVI depletion caused a significant decrease in the mean distance covered during the experiment when compared with untransfected and scrambled cells (Fig. 9b). An inhibitory effect of MVI depletion on myoblast migration was also observed in a wound-healing assay (not shown).

### MVI in myotube formation

**Fig. 10:**
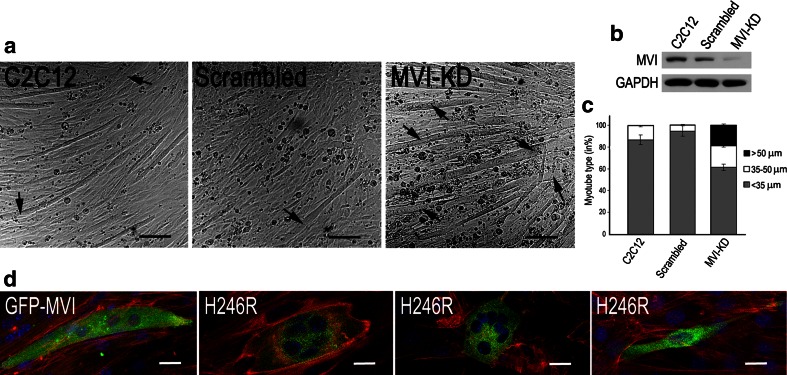
MVI in myotube formation. **a** Phase contrast of representative images of day-7 myotubes. Myotubes formed from untransfected myoblasts (C2C12), or from day-1 myoblasts transiently transfected with a scrambled construct or MVI shRNA (MVI-KD). *Arrows* point to very wide myotubes. **b** Representative Western blot analysis of MVI level at day 7. **c** Quantification of the number of wide myotubes in 12 random fields of view presented as % of all myotubes visible within the respective field of view. The values are means ± SD; ***p < 0.001. **d** Day-5 myotubes were transfected with pEGFP constructs encoding full-length human MVI fused with GFP (GFP-MVI) or the H246R MVI mutant. F-actin (*red*) and nuclei (*blue*) were visualized with Alexa Flour 546-conjugated phalloidin and DAPI, respectively. *Bars* in (**a**), 100 μm and in (**d**), 20 μm

Since MVI depletion led to disorganization of actin cytoskeleton and focal adhesions, and affected the organization of Golgi and ER, we hypothesized that these effects could influence the ability of MVI-depleted cells to form differentiated myotubes. To test this hypothesis, we transfected differentiating C2C12 myoblasts with scrambled shRNA or a construct targeting MVI and analyzed differentiated myotubes at day 7 after induction of myoblast fusion (Fig. 10a). Myotubes depleted in MVI were significantly wider than control cells or cells transfected with scrambled shRNA (Fig. 10a). To verify the depletion of MVI in these experiments, we lysed myotubes at day 7 post-fusion and confirmed reduced level of MVI protein using Western blot analysis (Fig. 10b).

To quantify the effect of MVI depletion on the tube morphology, we analyzed the thickness of myotubes from 12 images collected randomly (more than 150 myotubes for each condition were analyzed in two independent experiments). In untreated myotubes and myotubes transfected with scrambled shRNA, most myotubes were <35 μm wide with small percent of tubes reaching 35–50 μm in diameter (Fig. 10c). In contrast, about 20 % of MVI-depleted cells were more than 50 μm wide, and noteworthy, such wide myotubes were not observed in control cells (Fig. 10c). The MVI-KD myotubes were on average about twice as wide (54.4 ± 24.6 μm) as those formed by untreated (30.5 ± 8.0 μm) and scrambled (26.8 ± 5.4 μm) cells.

To further explore the notion that MVI could play a role in myoblast differentiation, we transfected day-5 myoblasts with the H246R MVI mutant or the original GFP-MVI construct as a control. Cells overexpressing the MVI mutant for 48 h did not form regular myotubes (Fig. 10d, central images). Instead, they became oval with centrally located nuclei in a nest-like structure, indicative of impairment in the nuclei positioning and/or cytoskeleton organization. Occasionally, thin myotube-like forms were spotted (Fig. 10d, right image). This is in contrast to control myotubes expressing wild-type MVI, which did not differ from their untransfected counterparts (Fig. 10d, left image).

## Discussion

In this study, we have addressed the role of MVI in two types of myogenic cells: C2C12 myoblasts as well as neonatal rat cardiomyocytes. Our data indicate that in myogenic cells, MVI may be involved in the organization of actin cytoskeleton, Golgi and endoplasmic reticulum, in cell migration and adhesion, in the development of the neuromuscular junction postsynaptic machinery as well as in myotube formation.

### Distribution and expression of MVI in myogenic cells

MVI level decreased during myoblast differentiation into myotubes, similarly to several other unconventional myosins present in myogenic cells (Wells et al. 1997). We also observed differences in MVI splice variant expression pattern between cells at different stages of differentiation. During differentiation, the levels of MVI variants with large (LI) and small inserts (SI) decreased, and the predominant variant was the one without inserts, thus resembling the pattern characteristic for adult rat skeletal muscles and non-polarized cells (Karolczak et al. 2013; Aschenbrenner et al. 2003; Au et al. 2007; Dance et al. 2004; Warner et al. 2003). To our knowledge, this is the first study to address MVI variant expression during cell differentiation. Furthermore, as the differentiation was progressing, MVI localization was changing from the nearly uniformly cytoplasmic to more peripheral. MVI localization was especially prominent at edges of myotubes in vicinity to areas of increased F-actin concentration.

### MVI in ER and Golgi organization

The observation that MVI is localized next to ER markers, especially in myotubes, is in line with our earlier observation of its presence within the ER of neurosecretory PC12 cells and sarcoplasmic reticulum (SR) of mature striated muscles (Karolczak et al. 2013; Majewski et al. 2010). It was previously shown that MVI depletion significantly affects Golgi organization (Warner et al. 2003). The cisternae are smaller and more fragmented in fibroblasts from mice lacking MVI (*Snell’s waltzer* model) or PC12 cells with MVI knockdown (Warner et al. 2003; Majewski et al. 2011). Also, our observation that the Golgi cisternae are more compact in the cells overexpressing H246R mutant indicates that MVI motor activity seems to be important for proper organization of this compartment. Further studies on the mutant motor activity are yet required.

A decrease in the ER area in MVI-KD myoblasts indicates that MVI could be involved not only in Golgi and but also in ER organization. Further supporting this notion, we saw differentiation-dependent colocalization of MVI with SERCA in rat neonatal cardiomyocytes. We are currently working to decipher the precise role of MVI in intracellular membrane organization by identifying MVI-interacting proteins.

### MVI involvement in myoblast migration and cell adhesion

Our observation that depletion of MVI inhibited myoblast migration is in agreement with numerous studies, including those on neurosecretory PC12 cells as well as on Drosophila border or ovarian cancer cells (Geisbrecht and Montell 2002; Yoshida et al. 2004; Majewski et al. 2011). The data presented here indicate that this inhibitory effect could result from the involvement of MVI in the regulation of actin organization and impairment of formation of adhesion structures. We demonstrate that MVI depletion leads to a decrease in the size of vinculin-containing adhesion complexes, which indicates that MVI could be involved in regulation of the size of focal adhesions (Zaidel-Bar et al. 2003). Also, we observed a possible interaction of MVI with talin as well as a correlation between MVI and talin expression levels both in undifferentiated myoblasts and mature myotubes. These findings are consistent with the hypothesis of Maddugoda et al. (2007) that MVI plays a critical role in regulation of the morphogenesis of the cell–substratum (or cell–cell) contacts. Interestingly, interaction of talin with *Dictyostelium discoideum* myosin VII, another unconventional myosin expressed in all Metazoa, was reported to play an important role in adhesion complex dynamics (Tuxworth et al. 2005; Galdeen et al. 2007).

### MVI in neuromuscular junction development

MVI was recently found in the postsynaptic region of the neuromuscular junction (NMJ) of rat hindlimb muscles and postulated to be involved in junction maintenance and/or neuromuscular transmission (Karolczak et al. 2013). Our data suggest that MVI might also play a role in the development of the postsynaptic part of the junction, since we observed enrichment of MVI in the regions corresponding to nascent acetylcholine receptor-rich clusters. This hypothesis is supported by the presence and engagement of MVI in synaptic transmission at the NMJ of the body wall muscle of the Drosophila third instar larvae (Kisiel et al. 2011, 2014). Further studies on *Snell’s waltzer* mice will shed further light on the involvement of MVI in NMJ function. Consistently with MVI function in synaptic transmission, MVI has been shown to be important for brain synapse function (Osterweil et al. 2005; Yano et al. 2006). Furthermore, depletion of another unconventional myosin (VA) caused severe fragmentation and size reduction in the NMJ, as well as impairment of acetylcholine receptors in the junction Roder et al. 2008, 2012), which further strengthens the idea that unconventional myosins may be important for neuromuscular transmission.

### MVI in myotube formation

Increasing evidence indicates that multiple cell signaling pathways play critical roles in myoblast fusion, including those involved in cytoskeleton organization, cell adhesion, and migration (Hindi et al. 2013). Our data show that MVI could be involved in myoblast differentiation, since MVI depletion in myoblasts caused the formation of significantly wider myotubes. Moreover, overexpression of the inactive H246R MVI mutant, previously associated with hypertrophic cardiomyopathy (Mohiddin et al. 2004), resulted in the formation of aberrant myotubes with centrally placed nuclei. These effects could be due to the above-described effects of MVI depletion on myoblast migration and adhesion but also could result from a defect in vesicle transport (Fig. 2e, f) or from impairment of nuclear positioning. MVI was previously shown to be required for targeted membrane transport during cytokinesis, which could also affect myotube formation and morphology (Arden et al. 2007). While the molecular mechanisms of the involvement of MVI in in vitro myotube formation are unknown, it is plausible that the observed effects could to some extent explain the hypertrophic cardiomyopathy phenotype reported in a patient with the H246R mutant and in MVI knockout mice (Mohiddin et al. 2004; Williams et al. 2013).

## Conclusions

The data described herein indicate that MVI plays important role in myogenic cells, also during their differentiation into myotubes. Since MVI is enriched within nascent acetylcholine receptor-rich clusters, we postulate that by providing the building blocks of postsynaptic machinery and maintaining them within the newly formed junction region, this molecular motor could participate in the neuromuscular junction development. The differentiation-dependent increase in the presence of MVI in the ER and changes in the ER organization evoked by its depletion imply also a role for MVI in the ER organization/function. We believe that similarly to observations made for the Golgi (Warner et al. 2003; Majewski et al. 2011), this could be related to the MVI involvement both in the actin cytoskeleton organization and in the vesicle trafficking. We postulate that MVI participates not only in a local transport within the ER membranous compartment but also in anchoring the ER membranes to the adjacent actin cytoskeleton (via its motor domain). Observation that MVI-depleted myoblasts have impaired migration and adhesion as well as form aberrant myotube indicates that MVI plays important role in myotube formation. Moreover, impairment of myotube formation was also observed in cells overexpressing MVI mutant with the motor domain. We postulate that interaction of MVI with talin, a crucial element of the adhesion structures, and involvement of MVI in the actin cytoskeleton organization are the key features that link this motor with myotube formation process. Further studies are needed to elucidate the molecular mechanisms of possible involvement of MVI in muscle differentiation. Particularly, a thorough examination of skeletal (and cardiac) muscle of the *Snell’s waltzer* mice that is missing to date should yield further insight into MVI function in muscle cells. We believe it may bring new information on MVI involvement in muscle functioning, similarly to the studies on a role for this molecular motor in the brain (Osterweil et al. 2005; Yano et al. 2006), kidney (Gotoh et al. 2010), and intestines (Ameen and Apodaca 2007), despite that no obvious symptoms were present at first.
